# Endophytic *Trichoderma* species from rubber trees native to the Brazilian Amazon, including four new species

**DOI:** 10.3389/fmicb.2023.1095199

**Published:** 2023-04-18

**Authors:** Vanessa Nascimento Brito, Janaina Lana Alves, Kaliane Sírio Araújo, Tiago de Souza Leite, Casley Borges de Queiroz, Olinto Liparini Pereira, Marisa Vieira de Queiroz

**Affiliations:** ^1^Faculdade de Ciências Biológicas, Universidade Federal do Pará, Altamira, Pará, Brazil; ^2^Laboratório de Genética Molecular de Microrganismos, Departamento de Microbiologia Agrícola, Universidade Federal de Viçosa, Viçosa, Minas Gerais, Brazil; ^3^Instituto Federal do Sudeste de Minas Gerais—Campus Juiz de Fora, Juiz de Fora, Minas Gerais, Brazil; ^4^Departamento de Fitopatologia, Universidade Federal de Viçosa, Viçosa, Minas Gerais, Brazil

**Keywords:** DNA barcoding, GCPSR, hevea, hypocreales, phylogeny, taxonomy

## Abstract

Fungi belonging to the genus *Trichoderma* have been widely recognized as efficient controllers of plant diseases. Although the majority of isolates currently deployed, thus far, have been isolated from soil, endophytic *Trichoderma* spp. is considered to be a promising option for application in biocontrol. In this study, 30 endophytic *Trichoderma* isolates—obtained from the leaves, stems, and roots of wild *Hevea* spp. in the Brazilian Amazon—were analyzed using specific DNA barcodes: sequences of internal transcribed spacers 1 and 2 of rDNA (ITS region), genes encoding translation elongation factor 1-α (TEF1*-α*), and the second largest subunit of RNA polymerase II (*RPB*2). The genealogical concordance phylogenetic species recognition (GCPSR) concept was used for species delimitation. A phylogenetic analysis showed the occurrence of *Trichoderma* species, such as *T*. *erinaceum*, *T*. *ovalisporum*, *T*. *koningiopsis*, *T*. *sparsum*, *T*. *lentiforme*, *T*. *virens*, and *T*. *spirale*. Molecular and morphological features resulted in the discovery of four new species, such as *T*. *acreanum* sp. nov., *T*. *ararianum* sp. nov., *T*. *heveae* sp. nov., and *T*. *brasiliensis* sp. nov. The BI and ML analyses shared a similar topology, providing high support to the final trees. The phylograms show three distinct subclades, namely, *T*. *acreanum* and *T*. *ararianum* being paraphyletic with *T*. *koningiopsis*; *T*. *heveae* with *T*. *subviride*; and *T*. *brasiliensis* with *T*. *brevicompactum*. This study adds to our knowledge of the diversity of endophytic *Trichoderma* species in Neotropical forests and reveals new potential biocontrol agents for the management of plant diseases.

## Introduction

The Amazon biome is recognized worldwide for its high biodiversity of flora and fauna. Thus, the intensification of research into the characterization of the microbial biodiversity found in this biome can provide useful information for a range of biotechnological strategies, including the production of enzymes (Pereira et al., 2015) and antimicrobial compounds (Nuankeaw et al., 2020).

The rubber trees *Hevea brasiliensis* (Willd. ex A.Juss.) Müll.Arg. and *Hevea guianensis* Aubl. are Euphorbiaceae species native to the Amazon rainforest. *Hevea brasiliensis* is the main source of raw materials for the production of natural rubber (Zhu et al., 2015; Meenakumari et al., 2018). Worldwide production, mainly concentrated in Southeast Asia, generates over 10 million tons per year, of which more than 80% is extracted by small producers in a chain that benefits approximately 20 million people (Rivano et al., 2013). However, fungal pathogens, such as *Pseudocercospora ulei* (Henn.) Hora & Mizubuti (formerly known as *Microcyclus ulei*), the causal agent of South American leaf blight of rubber (Hora Júnior et al., 2014), have been the major constraints to production in its Neotropical center of origin since the establishment of commercial rubber plantations.

The genus *Trichoderma* (*Hypocreales*) is cosmopolitan and ubiquitous in the environment, commonly being found in the microflora of natural and agricultural soils, although some taxa are also able to endophytically colonize plant tissues. *Trichoderma* species have now been isolated as endophytes from a range of woody plants, including *Herrania* sp. (Crozier et al., 2006), *Aegle marmelos* (L.) Corrêa (Gond et al., 2007), *Theobroma* spp. (Evans et al., 2003; Bae et al., 2009), *Hevea* spp. (Chaverri et al., 2010; Gazis and Chaverri, 2010), and *Coffea* spp. (Rodriguez et al., 2021). Moreover, this genus is widely recognized as a parasite of other fungi (Harman et al., 2004; Mukherjee et al., 2013; Rodriguez et al., 2021).

Numerous studies have been conducted on *Trichoderma* spp., with particular emphasis on mycoparasitic activities and their potential as biocontrol agents of plant diseases, including *Rhizoctonia solani* Kühn (Almeida et al., 2007), *Moniliophthora perniciosa* (Stahel) Aime & Phillips-Mora (De Souza et al., 2008), *Pyricularia oryzae* Cavara (Dey et al., 2013), *Phytophthora colocasiae* Racib. (Nath et al., 2014), *Phytophthora megakarya* Brasier & Griffin (Mbarga et al., 2014), *Phymatotrichopsis omnivora* (Shear) Hennebert (Guigón-López et al., 2015), *Sclerotium cepivorum* Berk. (Rivera-Méndez et al., 2020), and *Hemileia vastatrix* Berk. & Broome (Rodriguez et al., 2021). *Trichoderma* spp. are also known to produce enzymes and secondary metabolites applicable to industrial processes (Saravanakumar et al., 2016; Baldoni et al., 2020), to induce systemic resistance in plants (Vitti et al., 2016) and promote plant growth (Kuswinanti et al., 2015).

Defining species within the genus *Trichoderma* has proven to be challenging because of their complex and cryptic characteristics that lack distinguishable phenotypic traits (Jaklitsch et al., 2008; Jaklitsch, 2009). Moreover, various morphospecies such as *Trichoderma harzianum* Rifai represent species complexes, and the taxonomy of this complex was updated by Chaverri et al. (2015), who identified numerous new species. This study also indicated a tendency for habitat specialization; thus, species commonly isolated from soil tend not to be endophytes, and species isolated as endophytes tend not to be isolated from soils.

In several studies, morphological characteristics (stromata) and ecological factors, such as specific fungus/host relationships, have been included to improve the morphological concept and species delimitation (Taylor et al., 2000). Without the use of multilocus molecular phylogenetics, all of these species would have been identified as *T*. *harzianum*. New species, such as *Trichoderma amazonicum* (Chaverri & Gazis), have gradually been recognized within the complex based on subtle phenotypic or biological characteristics (Chaverri et al., 2010). The taxonomy of *Trichoderma* has substantially expanded through the use of molecular phylogeny (Druzhinina et al., 2006; Singh et al., 2020). *Trichoderma* spp. are now commonly described using a combination of morphological characteristics (asexual and sexual), cultural features, ecology, phylogenetic analysis, and the genealogical concordance phylogenetic species recognition (GCPSR) concept (Taylor et al., 2000; Cai and Druzhinina, 2021), including the species isolated in this study.

Some endophytic isolates belonging to the genus *Trichoderma* have recently been recognized as new species (Chaverri et al., 2010; Druzhinina, 2011; Rodriguez et al., 2021; Zheng et al., 2021). This may be the result of rapid speciation of the genus following adaptation to new ecological niches (Chaverri et al., 2010, 2015), or a likely trend is that these researchers were most intensely looking for endophytes.

Since *Hevea* spp. are the main sources of natural rubber (Zhu et al., 2015), endophytic *Trichoderma* spp. from these hosts may have potential applications for the biocontrol of the main rubber pathogens, especially *Pseudocercospora ulei* (Gazis and Chaverri, 2010; Hora Júnior et al., 2014), although few studies have investigated endophytic fungi in rubber trees (Gazis and Chaverri, 2010; Araújo et al., 2018, 2020). Thus, the aim of the present study was to increase our knowledge of *Trichoderma* endophytes in wild rubber, using morphological and molecular characterization to assess their biodiversity and potentially identify new species, as well as to generate information that could be used in future biotechnological strategies.

## Materials and methods

### Sample collection, processing, and growth conditions

Endophytic *Trichoderma* spp. were isolated from leaves, stems, and roots of *Hevea* spp. trees from natural populations in the Brazilian Amazon (Acre and Amazonas states). The collection has been detailed and described by Araújo et al. (2018, 2020), and fungal isolation was performed following the recommendations suggested by Wirsel et al. (2001), Evans et al. (2003), and Leite et al. (2013) and reported by Araújo et al. (2018, 2020). Currently, these isolates are kept in the collection of fungi at the Laboratory of Molecular Genetics of Microorganisms, BIOAGRO, Universidade Federal de Viçosa, MG, Brazil. A representative sample of each new species was deposited in the Herbarium, the Universidade Federal de Viçosa, (Herbarium VIC), and the isolates were deposited in the culture collection Coleção Octávio de Almeida Drumond (COAD), the Universidade Federal de Viçosa. The holotype is a dried culture of the endophytic fungus growing on potato dextrose agar (PDA).

### Morphological observations

The characterization of new species was determined by observations on different media, namely, PDA (500 mL of potato infusion prepared from 200 g of potatoes, 20 g of glucose, and 17 g of agar, and made up with 1 L with distilled water), cornmeal dextrose agar (CMD; Sigma—C1176), and, synthetic nutrient-poor agar (SNA; Nirenberg, 1976). To monitor colony growth rate, CMD, SNA, and PDA plates were incubated at 25, 30, and 35°C, with a 12 h light/dark cycle for 72 h. Photomicrographs and biometric descriptions of the reproductive structures of the isolates were obtained from microculture on SNA, incubated for 24–48 h at 25°C with a 12 h light/dark cycle. Slides were mounted in lacto-glycerol, the fungal structures were examined, and the images were produced using a light microscope (Olympus BX50 and BX53), fitted with a Q-Color 3 digital camera (Olympus PM-C35DX), together with Q-Capture Pro 6 software. Conidiophores and conidia were also measured using ImageJ software (Abramoff et al., 2004). To monitor colony growth rate assays, 5-mm mycelial disks were placed in 9 cm-diameter Petri plates containing 20 mL of PDA, CMD, or SNA media. The plates were cultured for up to 7 days for all assessments, and the mycelial diameter was measured using a caliper. Each culture medium was tested in triplicate.

### DNA extraction

Isolates were grown on PDA for 5 days under the conditions described earlier. Approximately 50 mg of mycelium was scraped from the colonies formed on the plates and placed in sterile tubes. A Wizard® Genomic DNA Purification Kit (Promega, Madison, WI, United States) was used according to the manufacturer’s instructions.

### PCR, sequencing, and phylogenetic analysis

For analysis by PCR and sequencing, the region of nuclear rDNA containing the internal transcribed spacer regions 1 and 2 and the 5.8S rDNA gene region was amplified using the ITS1 and ITS4 primers (White et al., 1990). Translation elongation factor 1-α (TEF1*-α*) was amplified using ef1–728F and LLErev primers (Carbone and Kohn, 1999; Jaklitsch et al., 2005), and RNA polymerase II subunit B (*RPB*2) was amplified using fRPB2–5 fl and fRPB2–7cR primers (Liu et al., 1999; Table 1). PCR conditions are presented in Table 1. Amplification products were subjected to 1.2% agarose gel electrophoresis and analyzed. The resulting products were purified and sequenced by Macrogen, South Korea.^1^ The sequences obtained were edited and corrected manually using the DNA BASER sequence assembly software system^2^ and compared by a sequence similarity search with the GenBank database using the algorithm for local alignment of nucleotide sequences (Blastn; Altschul et al., 1990) and TrichoBLAST (Kopchinskiy et al., 2005), which allowed the identification of isolates as belonging to species of *Trichoderma* or just to the genus. Sequence alignments were performed using MUSCLE implemented in MEGA6 (Tamura et al., 2013). The alignments of concatenated TEF1-α and *RPB*2 sequences were manually adjusted, contained 128 sequences of species of the genus *Trichoderma*, 30 of which were isolated in the course of this study, and 98 isolates retrieved from GenBank, including the two sequences of the outgroup (Table 2). In total, the dataset comprised 119 partial TEF1-α sequences and 112 partial *RPB*2 sequences. The NEXUS file was interleaved using Paup 4b10 software (Swofford et al., 2001) to verify whether it was possible to concatenate the amplified TEF1-α and *RPB*2 sequences. Individual phylogenetic analysis of TEF1-α and *RPB*2 and concatenated multilocus analysis of all analyzed regions were carried out by Bayesian inference (BI) using MrBayes 3.2.1 (Huelsenbeck and Ronquist, 2001). The best evolutionary models were selected according to the Akaike Information Criterion (AIC) using MrModeltest v2.3 program (Nylander, 2004). Nucleotide substitution models in the single and multilocus trees were GTR + I + G for TEF1-α and SYM + I + G for *RPB*2. For all trees, the BI was estimated in the CIPRES Science Gateway Platform using Mr. Bayes 3.2.6. Two independent runs with four Markovian chain Monte Carlo (MCMC) procedures were conducted for 10 million generations, and the trees were sampled and retained every 1,000th generation. The first million tree samples were discarded in the burning phase, and the trees were summarized to generate a majority-rule consensus tree.

**Table 1:** PCR primers and conditions used for DNA amplification.

Gene/locus	Primer	Sequence (5′–3′)	PCR conditions	References
ITS	ITS 1	TCCGTAGGTGAACCTGCGG	3 min at 95°C; 36 cycles of 1 min at 95°C, 1 min at 51°C and 1 min at 72°C; and 7 min at 72°C	White et al. (1990)
	ITS 4	TCCTCCGCTTATTGATATGC		White et al. (1990)
TEF1-α	ef1–728F	TCCGTAGGTGAACCTGCGG	3 min at 95°C; 36 cycles of 1 min at 95°C, 1 min at 61°C and 1 min at 72°C; and 7 min at 72°C	Carbone and Kohn (1999)
	LLErev	TCCTCCGCTTATTGATATGC		Jaklitsch et al. (2005)
*RPB*2	fRPB2–5 fl	GAYGAYMGWGATCAYTTYGG	2 min at 95°C; 36 cycles of 1 min at 95°C, 1 min at 52°C to each second with increment of 0.2°C and 1 min at 72°C; and 7 min at 72°C	Liu et al. (1999)
	fRPB2–7cR	CCCATRGCTTGYTTRCCCAT		Liu et al. (1999)

**Table 2:** Strains and NCBI GenBank accession numbers.

Clade	Species	Strain	Country	Genbank access		
				ITS	TEF1-α	*RPB*2
*Viride*	*Trichoderma adaptatum*	HMAS:248801	China	**–**	KX428027	KX428045
*Viride*	*Trichoderma adaptatum*	HMAS:248800	China	**–**	KX428024	KX428042
*Viride*	*Trichoderma atroviride*	CBS 142.95	Slovenia	AY380906	AY376051	EU341801
*Viride*	*Trichoderma atroviride*	DAOM 222144		AF456916	AF456889	FJ442754
*Viride*	*Trichoderma atroviride*	CBS:119499		FJ860726	FJ860611	FJ860518
*Viride*	*Trichoderma paratroviride*	CBS:136489	Spain	**–**	KJ665627	KJ665321
*Viride*	*Trichoderma paratroviride*	S489	Spain	**–**	KJ665628	KJ665322
***Viride***	***Trichoderma* sp**.	**432F8C-AM**	**Brazil**	**MK713510**	**MT364219**	**–**
*Viride*	*Trichoderma subviride*	HMAS:273762	China	**–**	KU529132	KU529143
*Viride*	*Trichoderma subviride*	HMAS:273761		**–**	KU529131	KU529142
*Viride*	*Trichoderma beijingense*	HMAS:248804		**–**	KX428025	KX428043
*Viride*	*Trichoderma beijingense*	HMAS:248805		**–**	KX428026	KX428044
*Viride*	*Trichoderma caribbaeum*	CBS:119093		NR166015	KJ665443	KJ665246
*Viride*	*Trichoderma istrianum*	S123		**–**	KJ665521	KJ665280
*Viride*	*Trichoderma albofulvopsis*	HMAS:273760		**–**	KU529127	KU529138
*Viride*	*Trichoderma ochroleucum*	CBS:119502	United States	NR134401	FJ860659	FJ860556
*Viride*	*Trichoderma intricatum*	GJS 02-78	United States	EU264002	EU248630	EU241505
***Viride***	***Trichoderma* sp**.	**4F9R-AC**	**Brazil**	**MK026984**	**MT337600**	**MT322319**
***Viride***	***Trichoderma* sp**.	**26F9R-AC**	**Brazil**	**MK713508**	**MT337601**	**MT322320**
***Viride***	***Trichoderma* sp**.	**508F9R-AC**	**Brazil**	**MK713511**	**MT337599**	**MT322321**
*Viride*	*Trichoderma ovalisporum*	DIS 70A	United States	AY380897	AY376037	FJ442742
*Viride*	*Trichoderma ovalisporum*	DIS 203C	Brazil	DQ315458	DQ307540	FJ442796
*Viride*	*Trichoderma ovalisporum*	GJS 04-113	Vietnã	FJ442614	FJ463281	FJ442781
***Viride***	***Trichoderma acreanum* sp**.**nov**.	**COAD 3342**	**Brazil**	**MK713509**	**MT336736**	**MT322313**
***Viride***	***Trichoderma acreanum* sp**.**nov**.	**COAD 3343**	**Brazil**	**MK713501**	**MT336735**	**MT322311**
***Viride***	***Trichoderma acreanum* sp**.**nov**.	**COAD 3344**	**Brazil**	**MK713503**	**MT336737**	**MT322312**
***Viride***	***Trichoderma acreanum* sp**.**nov**.	**COAD 3345**	**Brazil**	**MK713505**	**MT327806**	**MT322314**
***Viride***	***Trichoderma acreanum* sp**.**nov**.	**COAD 3346**	**Brazil**	**–**	**MT327807**	**MT322317**
***Viride***	***Trichoderma acreanum* sp**.**nov**.	**COAD 3347**	**Brazil**	**MK713515**	**MT327809**	**MT322316**
***Viride***	***Trichoderma acreanum* sp**.**nov**.	**COAD 3348**	**Brazil**	**MK713507**	**MT327808**	**MT322315**
***Viride***	***Trichoderma* sp**.	**619F6C-AM**	**Brazil**	**MK713517**	**MT327810**	**–**
***Viride***	***Trichoderma* sp**.	**815F11R-AM**	**Brazil**	**MK713520**	**MT336733**	**MT322318**
*Viride*	*Trichoderma koningiopsis*	DAOM 222105		AY380901	AY376042	EU341810
*Viride*	*Trichoderma koningiopsis*	DIS 374A	United States	FJ442213	FJ463288	FJ442730
*Viride*	*Trichoderma koningiopsis*	GJS 04-199	Peru	FJ442654	FJ463268	FJ442789
*Viride*	*Trichoderma koningii*	S227		**–**	KC285596	JN715609
*Viride*	*Trichoderma tardum*	HMAS:248798	China	**–**	KX428020	KX428038
*Viride*	*Trichoderma tardum*	HMAS:248799	China	**–**	KX428021	KX428039
*Viride*	*Trichoderma bifurcatum*	HMAS:248795	China	**–**	KX428018	KX428036
***Viride***	***Trichoderma* sp**.	**20F5C-AM**	**Brazil**	**MK713500**	**–**	**MT322326**
***Viride***	***Trichoderma* sp**.	**610F5C-AM**	**Brazil**	**MK713516**	**MT336734**	**MT322325**
*Viride*	*Trichoderma erinaceum*	DAOM 230019	Thailand	DQ083009	**–**	KJ842151
*Viride*	*Trichoderma erinaceum*	DIS 7	Peru	DQ109534	DQ109547	**–**
*Viride*	*Trichoderma erinaceum*	GJS 02-103	Canada	KR873100	KR873097	KR873099
*Viride*	*Trichoderma sinokoningii*	HMAS:271397	China	**–**	KU529130	KU529141
*Viride*	*Trichoderma austrokoningii*	CBS:119080	Canadá	**–**	KJ871163	**–**
*Viride*	*Trichoderma austrokoningii*	CBS:119092	**–**	**–**	**–**	KJ842161
*Viride*	*Trichoderma austrokoningii*	CBS 247.63	United States	DQ315470	DQ307568	FJ442772
*Viride*	*Trichoderma vulgatum*	HMAS:248796	**–**	**–**	KX428019	KX428037
*Viride*	*Trichoderma vulgatum*	HMAS:248797	China	**–**	KX428035	KX428053
***Viride***	***Trichoderma heveae* sp**.**nov**.	**COAD2323**	**Brazil**	**MG751189**	**MT364220**	**MT322322**
***Viride***	***Trichoderma heveae* sp**.**nov**.	**COAD2645**	**Brazil**	**MK713518**	**MT364221**	**MT322323**
***Viride***	***Trichoderma heveae* sp**.**nov**.	**COAD2646**	**Brazil**	**MK713519**	**MT364222**	**MT322324**
*Viride*	*Trichoderma rogersonii*	GJS 98-77	United States	DQ323414	DQ307572	**–**
*Viride*	*Trichoderma rogersonii*	GJS 04-157	United States	DQ323415	DQ307558	JN133566
*Viride*	*Trichoderma rogersonii*	CBS:119503		FJ860826	FJ860690	FJ860583
*Viride*	*Trichoderma mangshanicum*	HMAS:248810		**–**	KX428032	KX428050
*Viride*	*Trichoderma mangshanicum*	HMAS:248811		**–**	KX428033	KX428051
*Viride*	*Trichoderma mangshanicum*	HMAS:248812		**–**	KX428034	KX428052
***Viride***	***Trichoderma* sp**.	**22F20C-AC**	**Brazil**	**MG751240**	**MT364218**	**MT327805**
*Viride*	*Trichoderma sparsum*	HMAS:273759	China	**–**	KU529136	KU529147
*Viride*	*Trichoderma subeffusum*	CBS:120929		NR134416	FJ860707	FJ860597
*Viride*	*Trichoderma strigosellum*	GJS 05-02		EU263997	EU248631	EU248607
*Viride*	*Trichoderma strigosellum*	DAOM 229937		EU280139	EU280030	KJ842147
*Viride*	*Trichoderma strigosellum*	DAOM 230018		FJ442649	FJ463279	**–**
*Viride*	*Trichoderma strigosum*	DAOM 166121		EU280120	EU280019	AF545556
*Viride*	*Trichoderma eijii*	HMAS:266642	China	KJ783301	KJ634769	KJ634736
*Viride*	*Trichoderma pezizoides*	CBS:115283	United States	NR138436		**–**
*Viride*	*Trichoderma pezizoides*	GJS 01-257	United States	**–**	AY937438	EU248608
*Viride*	*Trichoderma flaviconidium*	GJS 99-49	Costa Rica	DQ023301	DQ020001	EU883557
*Viride*	*Trichoderma evansii*	DIS 341HI	Cameroon	EU883568	EU883566	EU883558
*Viride*	*Trichoderma evansii*	DIS 380A	Cameroon	EU856295	EU856320	FJ150785
*Brevicompactum*	*Trichoderma limonium*	HMAS:248754		**–**	KX066248	KX066260
*Brevicompactum*	*Trichoderma limonium*	HMAS:248751		**–**	KX066247	KX066259
*Brevicompactum*	*Trichoderma arundinaceum*	GJS 05-184		EU330933	EU338280	EU338308
*Brevicompactum*	*Trichoderma arundinaceum*	NRRL 3199	United States	EU330932	EU338279	EU338307
*Brevicompactum*	*Trichoderma turrialbense*	CBS:112445	Costa Rica	EU330945	EU338284	EU338321
*Brevicompactum*	*Trichoderma turrialbense*	BBA 72294		EU330944	EU338282	EU338320
***Brevicompactum***	***Trichoderma brasiliensis* sp**.**nov**.	**COAD 2324**	**Brazil**	**MK713497**	**MT300486**	**MT300488**
***Brevicompactum***	***Trichoderma brasiliensis* sp**.**nov**.	**COAD 2642**	**Brazil**	**MK713498**	**MT300487**	**MT300490**
***Brevicompactum***	***Trichoderma brasiliensis* sp**.**nov**.	**COAD 2643**	**Brazil**	**MK713499**	**MT300492**	**MT300489**
***Brevicompactum***	***Trichoderma brasiliensis* sp**.**nov**.	**COAD 2644**	**Brazil**	**MK713514**	**MT300493**	**MT300491**
*Brevicompactum*	*Trichoderma brevicompactum*	CBS:112443	PapuaNewGuinea	EU330943	EU338281	EU338319
*Brevicompactum*	*Trichoderma brevicompactum*	CBS:112447		EU330942	EU338300	EU338318
*Brevicompactum*	*Trichoderma protrudens*	DIS 119F	India	EU330946	EU338289	EU338322
*Brevicompactum*	*Trichoderma rodmanii*	CBS:121553	Austria	FJ860824	FJ860687	FJ860580
*Brevicompactum*	*Trichoderma rodmanii*	CPK 2852	Austria	FJ860825	FJ860688	FJ860581
*Brevicompactum*	*Trichoderma margaretense*	CPK 3127	Austria	FJ860741	FJ860625	FJ860529
*Brevicompactum*	*Trichoderma aurantioeffusum*	CPK 3119	Austria	FJ860730	FJ860614	FJ860521
*Brevicompactum*	*Trichoderma aurantioeffusum*	CBS:119284	Austria	NR134383	FJ860613	FJ860520
*Brevicompactum*	*Trichoderma aurantioeffusum*	S565		**–**	KJ665430	**–**
*Brevicompactum*	*Trichoderma grande*	HMAS:273788	China	**–**	KX066255	**–**
*Brevicompactum*	*Trichoderma grande*	HMAS:248749	China	**–**	KX066254	KX066266
*Harzianum*	*Trichoderma breve*	HMAS:248844	China	KY687927	KY688045	KY687983
*Harzianum*	*Trichoderma breve*	HMAS:248845	China	KY687928	KY688046	KY687984
***Harzianum***	***Trichoderma* sp**.	**518F1C-AM**	**Brazil**	**MK713512**	**MT337597**	**MT311143**
***Harzianum***	***Trichoderma* sp**.	**765F5C-AM**	**Brazil**	**MG751207**	**MT955363**	**MT311145**
***Harzianum***	***Trichoderma* sp**.	**247F12R-AC**	**Brazil**	**MK713504**	**MT337598**	**MT311142**
*Harzianum*	*Trichoderma lentiforme*	DIS 218E	United States	FJ442220	FJ463310	FJ442793
*Harzianum*	*Trichoderma lentiforme*	DIS 173F	United States	FJ442253	FJ463347	FJ442787
*Harzianum*	*Trichoderma bannaense*	HMAS:248840	United States	NR154570	KY688037	KY687979
*Harzianum*	*Trichoderma bannaense*	HMAS:248865	China	KY687948	KY688038	KY688003
*Harzianum*	*Trichoderma azevedoi*	CEN1403	Brazil	MK714880	MK696638	MK696800
*Harzianum*	*Trichoderma azevedoi*	CEN1422	Brazil	MK714902	MK696660	MK696821
*Harzianum*	*Trichoderma afarasin*	DIS 377A	United States	FJ442665	FJ463322	FJ442799
*Harzianum*	*Trichoderma afarasin*	DIS 314F	United States	FJ442259	FJ463400	FJ442778
*Harzianum*	*Trichoderma endophyticum*	DIS 220 J	United States	FJ442254	FJ463330	FJ442690
*Harzianum*	*Trichoderma endophyticum*	DIS 220 K	United States	FJ442270	FJ463328	FJ442765
*Harzianum*	*Trichoderma endophyticum*	DIS 221E	United States	FJ442255	FJ463316	FJ442775
*Harzianum*	*Trichoderma camerunense*	GJS 99-230	United States	NR137300	AF348107	**–**
*Harzianum*	*Trichoderma camerunense*	GJS 99-231	United States	AY027783	AF348108	**–**
***Harzianum***	***Trichoderma* sp**.	**23F18C-AC**	**Brazil**	**MK713502**	**MT337596**	**MT311146**
***Harzianum***	***Trichoderma* sp**.	**549F18C-AC**	**Brazil**	**MK713513**	**MT337595**	**MT311144**
*Harzianum*	*Trichoderma harzianum*	DIS 218F	Ecuador	FJ442246	FJ463326	FJ442722
*Harzianum*	*Trichoderma harzianum*	DIS 219F	Ecuador	FJ442247	FJ463325	FJ442797
*Harzianum*	*Trichoderma harzianum*	DIS 221D	United States	FJ442248	FJ463389	FJ442794
***Strictipile***	***Trichoderma* sp**.	**597F2C-AC**	**Brazil**	**MG751249**	**MT311149**	**MT311147**
*Strictipile*	*Trichoderma spirale*	DIS 114I	United States	FJ442666	FJ463290	FJ442753
*Strictipile*	*Trichoderma spirale*	DIS 151E	United States	FJ442230	FJ463374	FJ442766
*Strictipile*	*Trichoderma spirale*	COAD2404	Cameroon	**–**	MK044091	MK044184
*Strictipile*	*Trichoderma spirale*	COAD2408	Cameroon	**–**	MK044096	MK044189
*Strictipile*	*Trichoderma spirale*	COAD2413	Cameroon	**–**	MK044105	MK044198
*Strictipile*	*Trichoderma spirale*	DAOM 183974	United States	NR077177	EU280049	AF545553
***Virens***	***Trichoderma* sp**.	**84F15C-AM**	**Brazil**	**MG751276**	**MT311148**	**MT311141**
*Virens*	*Trichoderma virens*	DIS 162	United States	FJ442669	FJ463367	FJ442696
*Virens*	*Trichoderma virens*	GJS 01-287	United States	DQ083023	AY750894	EU341804
*Virens*	*Trichoderma neocrassum*	DAOM 164916	United States	NR134370	AF534615	AF545542
*Virens*	*Trichoderma neocrassum*	GJS 95-157	United States	**–**	AF534602	AF545543
**Outgroup**	***Protocrea pallida***	**CBS:121552**	**Austria**	**EU703922**	**EU703897**	**EU703944**
**Outgroup**	***Protocrea pallida***	**CBS 299**.**78**	**Austria**	**NR111329**	**EU703900**	**EU703948**

(−) DNA sequence not found in data.bases. The bold values provided in table refer to isolates used in this study.

For maximum likelihood (ML) analyses, the concatenated tree with the two genes was generated in Sequence matrix v1.8125 (Vaidya et al., 2011). The ML analyses for single-loci trees and multilocus were estimated in the CIPRES Science Gateway Platform using RaxML-HPC v.8 (Stamatakis, 2006). For the concatenated dataset, all free modal parameters were estimated by RAxML with an ML estimate of 25 per site rate category. The concatenated dataset was partitioned by loci in the RAxML platform. The RAxML software accommodated the GTR model of nucleotide substitution with the additional options of modeling rate heterogeneity (G) and proportion invariable sites (I).

Phylogenetic trees (BI and ML) were visualized using FigTree.^3^ In BI, tree clades with posterior probability values below 95% were excluded (Harada et al., 1995; Rannala and Yang, 1996), and bootstrap values (≥70%) are given near nodes. The topologies obtained using the two methods (BI and ML) were then compared, and the phylogram layout was edited with CorelDRAW Graphics Suite 2020.

### Genealogical concordance phylogenetic species recognition

The genealogical concordance phylogenetic species recognition (GCPSR) concept was applied to identify independent evolutionary lineages using the tools developed in Perl language by Brankovics et al. (2017) available at GitHub.^4^ The phylogenetic trees were built side-by-side using the ggtree package in R 4.0 (R Core Team 2021), to highlight the patterns of genealogical concordance and non-concordance.

## Results

A total of 30 *Trichoderma* isolates were obtained from leaf, stem, and root samples of *Hevea* spp. in the Brazilian Amazon Forest. In quantitative terms, 13 from the state of Acre and 17 from the state of Amazonas were isolated (Araújo et al., 2018, 2020). The preliminary sequence similarity search with the ITS region and TEF1-α and *RPB*2 gene sequences showed that the 30 isolates belong to the *Brevicompactum*, *Harzianum*, *Virens*, *Viride*, and *Strictipile* clades. Therefore, as a next step, a phylogenetic analysis was conducted using a single locus of ITS, TEF1-α, and *RPB*2, and a multilocus dataset of concatenated TEF1-α and *RPB*2, which revealed the presence of seven different *Trichoderma* species. Isolates 20F5C–AM and 610F5C–AM were phylogenetically close to *Trichoderma erinaceum* (Bissett, Kubicek, and Szakacs), the isolates 619F6C–AM and 815F11R–AM were phylogenetically close to *Trichoderma koningiopsis* (Samuels, Suárez, and Evans), and 4F9R–AC, 26F9R–AC, and 508F9R–AC were phylogenetically close to *Trichoderma ovalisporum* (Samuels and Schroers). In addition, in the *Viride* clade, isolate 22F20C–AC was close to *T*. *sparsum*, and 432F8C–AM was close to *Trichoderma atroviride* Karst. In the *Harzianum* clade, isolates 23F18C–AC and 549F18C–AC were close to *Trichoderma azevedoi* (Valadares-Inglis & Inglis), 518F1C–AM and 765F5C–AM were close to *Trichoderma breve* (Chen & Zhuang), and 247F12R–AC was close to *Trichoderma lentiforme* (Rehm; Chaverri, Samuels & Rocha). The isolate 597F2C–AC was phylogenetically close to *T*. *spirale* Bissett (*Strictipile* clade) and 84F15C–AM was close to *Trichoderma virens* (Miller, Giddens & Foster) Arx. (*Virens* clade). These *Trichoderma* spp. were retained for future research to confirm species-level classification. In addition to the molecular data, the four new species were characterized morphologically, such as *Trichoderma acreanum* sp. nov., *Trichoderma ararianum* sp. nov., *Trichoderma heveae* sp. nov., and *Trichoderma brasiliensis* sp. nov. Phylogenetic trees and DNA sequence alignment data were deposited in TreeBase (Study 29026).

**Figure 1:**
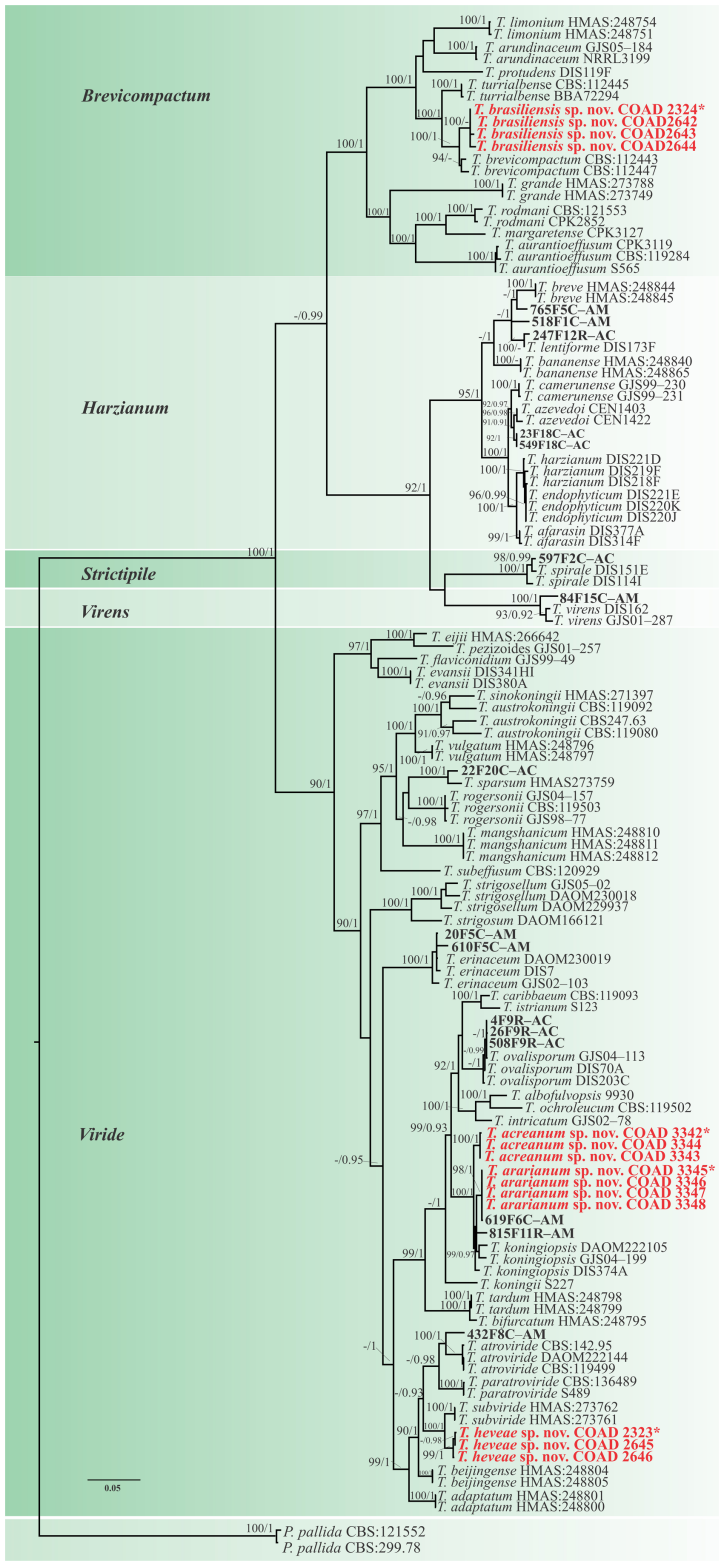
Phylogenetic tree of *Trichoderma* spp. based on combined *RPB*2 and TEF1-α sequence datasets. Bootstrap values (≥70%) of the ML analyses, as well as posterior probability scores (≥0.95) from a BI of the same dataset, are indicated at well-supported nodes together with thickened branches. The scale bar represents the number of expected substitutions per site (0.05). The isolates belonging to known species obtained in this study are in bold red. Isolates of new species described in this study are in bold red. Holotype labeled with an asterisk. The tree is rooted in two isolates of *Protocrea pallida* (CBS:121552 and CBS:299.78).*”–”* indicates a lack of support.

The clade classification was strongly supported by bootstrap and posterior probability values (Figure 1). The three single loci are shown in Supplementary Figures 1–3. *Viride* was the clade with the highest number and percentage of isolates (19), accounting for 63.3% of the total. The *Harzianum* clade included five and the *Brevicompactum* clade comprised four isolates, accounting for 16.7 and 13.3%, respectively. *Strictipile* and *Virens* clades were represented by one isolate each (3.3%).

**Figure 2:**
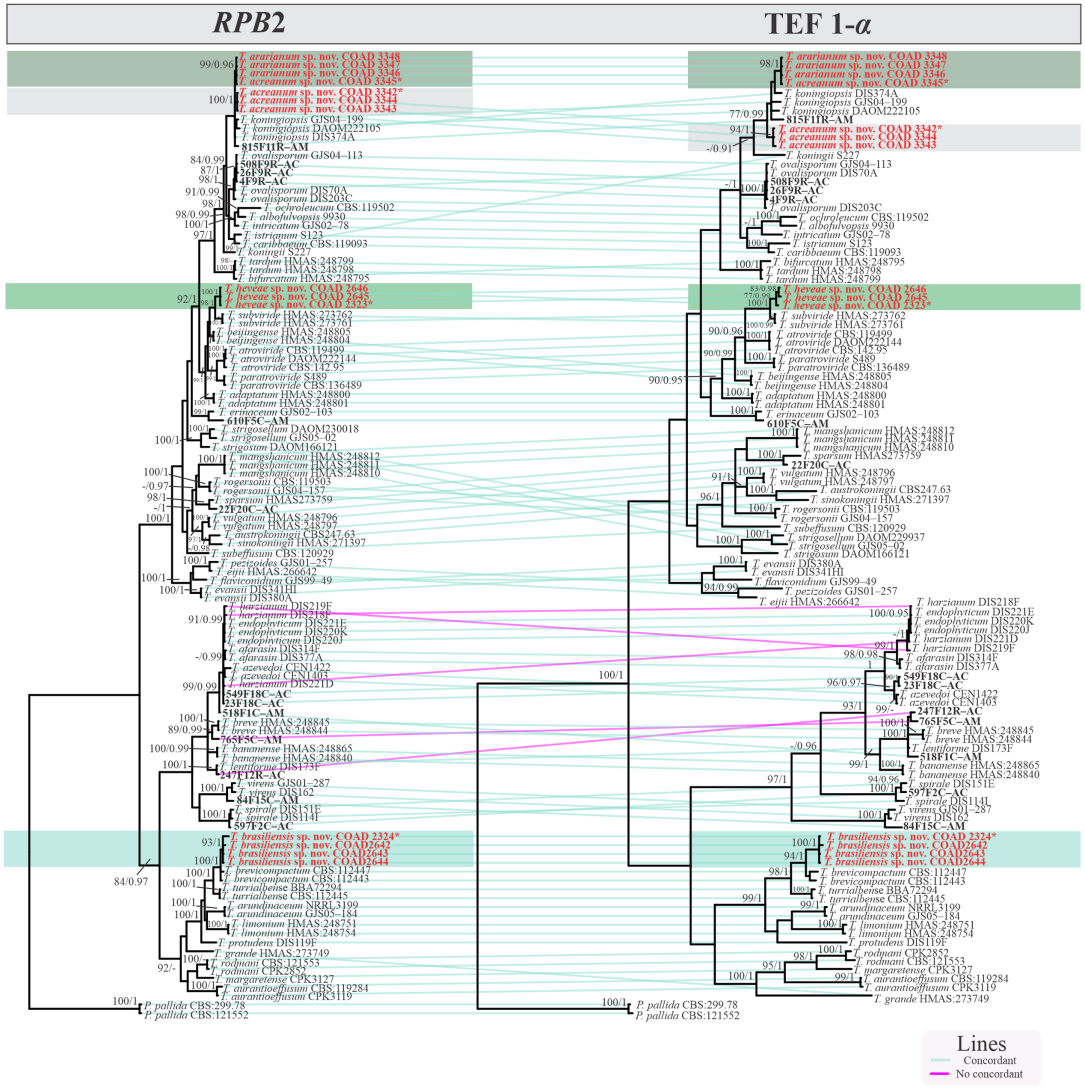
Phylogram GCPSR based on TEF1*-*α and *RPB*2 sequence datasets. Simultaneous analysis of two gene genealogies to show the transition from concordance among branches used to diagnose species.

Isolates were recognized in their respective clades based on the previously accepted GCPSR concept (Taylor et al., 2000; Dettman et al., 2003). Thus, by applying genealogical concordance, the clade was present in the majority of the single-locus topologies (TEF1-α and *RPB*2), as revealed by the multilocus tree. In contrast, genealogical non-discordance (or discordance) was not observed, since a clade must be well supported, as judged by BI and ML, by at least one single-locus genealogy and not contradicted in any other single-locus topology (Figure 2).

A total of three new species were identified in the *Viride* clade, such as *T*. *acreanum* sp. nov. (COAD3345), *T*. *ararianum* sp. nov. (COAD3342), and *T*. *heveae* sp. nov. (COAD2323). The other six isolates were identified as *Trichoderma* sp. and formed a monophyletic clade with strong statistical support. Five isolates (23F18C–AC, 549F18C, 247F12R–AC, 765F5C–AM, and 518F1C–AM) were included in the *Harzianum* clade and were not conclusively characterized to the species level.

Conversely, in the *Brevicompactum* clade, the new taxon, *T*. *brasiliensis*, represented by four isolates (COAD2324, COAD2642, COAD2643, and COAD2644) and was clearly distinguished by strong support from the other clade species.

Of the 30 isolates, 19 were isolated from stems. They were identified as *T*. *heveae* sp. nov. (three isolates), *T*. *acreanum* sp. nov. (three isolates), *T*. *ararianum* sp. nov. (four isolates), and *Trichoderma* sp. (11 isolates) present in all clades. A total of nine were isolated from roots, and they were identified as *T*. *brasiliensis* sp. nov. (four isolates) and *Trichoderma* sp. (five isolates). *Trichoderma brasiliensis* sp. nov. was found exclusively in the roots of *H*. *brasiliensis* in Amazonas. In contrast, two isolates were found in the leaves of *H*. *brasiliensis* and *H*. *guianensis* and are proposed here as a new species, *T*. *heveae* sp. nov.

## Taxonomy

**Figure 3:**
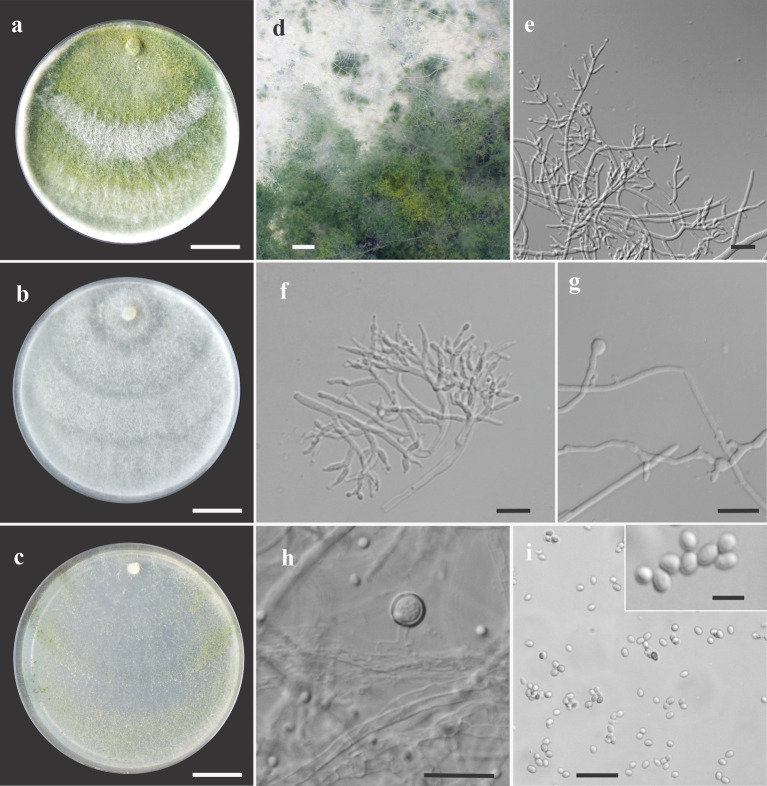
Asexual morph of *Trichoderma acreanum* sp. nov. from *Hevea brasiliensis* (VIC47506, holotype). (**A–C**) Colony appearances (PDA, CMD, and SNA; 7 days, 25°C). (**D**) Sporulating pustules (SNA). (**E**,**F**) Conidiophores and phialides (SNA). (**G**) Chlamydospore (after 48 h). (**H**) Chlamydospore (after 7 days). (**I**) Conidia. Scale bars: (**A–C**) 20 mm, (**D**) 0.2 mm, (**E**,**F**) 15 μm, (**G**) 10 μm, (**H**) 25 μm, (**I**) 20 μm (inside = 5 μm).

***Trichoderma acreanum*** V. N. Brito, J. L. Alves & M. V. Queiroz, **sp**. **nov**., Figure 3.

MycoBank MB830208

*Etymology*: “acreanum,” the specific epithet, refers to the state of Acre, Brazil.

*Holotype*: Brazil. State of Acre, Cruzeiro do Sul, Igarapé Campinas, coordinates 7°46′00.6″S, 72°15′03.3″W, K. S. Araújo, 26 July 2017 (holotype VIC47506). Ex-type culture of COAD3342.

*Sexual morph* not seen. Colonies fast growing at 25 and 30°C on all media; no growth at 35°C. No pigment or distinctive odor was observed on media PDA, SNA, and CMD. The radial growth rate on CMD was 77–79 mm at 25°C and 55–62.5 mm at 30°C, after 72 h. Colonies covering the plates after 4 days at 25°C; mycelium white, flat, and floccose in texture. After 7 days, some areas became dull green due to sporulation. Chlamydospores globose to irregular, terminal, intercalary, thick-walled, brown. Conidiophores comprising a main axis, and short fertile branches or phialides arising along the length of this axis, more or less paired with internodes, the terminal part of conidiophore sparingly branched. Phialides solitary or in diverging terminal whorls of 2–3, ampulliform, slightly inclined or curved in upper part, widest part mostly central, with a short neck, 6–11 μm × 2–3 μm (*n* = 30). Conidia yellow-green, smooth, globose to subglobose, 2–4 μm × 2.5–3 μm (*n* = 30). The radial growth rate on PDA was 78–80 mm at 25°C, 67.5–70 mm at 30°C, and 5–12 mm at 35°C, after 72 h. Colonies covered the plate after 4 days at 25°C; mycelium dense, texture floccose to velvety, mixed mycelia dark-herbage green, white, and grayish-yellow green. Chlamydospores terminal and intercalary, mostly globose, thick-walled, brown. On SNA, the radial growth rate was 42.5–45 mm at 25°C, 40–44 mm at 30°C, and 6–8 mm at 35°C, after 72 h. Colonies covered the plate after 6 days at 25°C; mycelia thin, floccose, and white, with discrete concentric zones due to sporulation, arranged in several irregular zones spreading from the center. Chlamydospores terminal and intercalary, brown, and globose.

Habitat and host range: endophytic in the stems of living wild *Hevea* spp. trees.

Additional specimens examined: Brazil. State of Acre, Xapuri, Reserve Chico Mendes, coordinates 10°49′59.1″S, 68°23′11.2″W, K. S. Araújo, 26 July 2017 (COAD3343, VIC47507); Brazil. State of Acre, Cruzeiro do Sul, Igarapé Campinas, coordinates 7°46′00.6″S, 72°15′03.3″W, K. S. Araújo, 26 July 2017 (COAD3344, VIC47508).

*Notes:* The concatenated tree with TEF1-α and *RPB*2 sequences clearly indicates that the three isolates belong to a new taxon (Figure 1). In addition, phylogenetic species were recognized based on two main previously accepted criteria genealogical concordance, wherein the clade (*T*. *acreanum*) was present in the two single-locus genealogies and Genealogical Non-Discordance, wherein the clade (*T*. *acreanum*) was well supported in the least one single-locus genealogy and was not contradicted in any other single-locus genealogy at the same level of support (Figure 2, Supplementary Figures 1, 2). *Trichoderma acreanum* sp. nov. is morphologically similar to several species of the *Trichoderma viride* Pers. clade (Samuels et al., 2006). In *Trichoderma acreanum* sp. nov., the phialides arise singly and are widely spaced directly from the main axis, which can have up to three phialides at its tip and is flask-shaped, straight, or slightly hooked; those forming at the tips of long conidiophores are often narrowly cylindrical or tapering uniformly from the base to the tip and swollen in the middle. However, in *T*. *koningiopsis*, the phialides are densely clustered with very short internodes, typically straight, hooked, or sinuous, narrowly lageniform, in whorls of 2–5, with several phialides at times arising from the same point and crowded. In addition, the conidia of the new taxon are shorter in length and subglobose compared with *T*. *koningiopsis* which has larger, ellipsoidal conidia (Table 3).

**Table 3:** Morphological differences relevant to the separation of new species of *Trichoderma* described as endophytes from *Hevea* spp. from the Amazon Forest.

Features	Species						
	*T*. *brevicompactum*	*T*. *subviride*	*T*. *koningiopsis*	VIC 44362	VIC 44363	VIC 47506	VIC 47509
Conidia (length–μm)	2.54 (± 0.18)	(2–) 2.5–3.8 (−4.5)	(3–) 3.5–4.5 (−6.2)	(2–) 3–4 (−4)	(2–) 3–3 (−4)	(2–) 2.5–3.5 (−4)	(2–) 2.5–4 (−5)
Conidia (width–μm)		(2–) 2.5–3.2	(2–) 2.2–3 (−3.5)	(2–) 3–3 (−4)	(2–) 3–3 (−4)	(2–) 2.5–2.5 (−3)	(2–) 2.5–3 (−3.5)
Phialide (length–μm)	5.50 (± 0.39)	(4.5–) 5–6.5 (−7)	(3.5–) 5.5–9.2 (−16)	(4–) 8–8 (−12)	(5–) 8–9 (−16)	(6–) 6.5–10 (−11)	(6–) 6.5–10 (−11)
Phialide (width–μm)	3.56 (± 0.24)	2–3	(2–) 2.7–3.5 (−4.5)	(2–) 3–3 (−4)	(2–) 3–3 (−4)	(2–) 2.5–2.5 (−3)	(2–) 2.5–2.5 (−3.5)
Conditions	Colony on PDA after 72 h (mm)
25°C/72 h		60–63	(45–) 51–63 (−67)	77.5	64.5–66.5	78–80	80–82.5
30°C/72 h			(20–) 52–72	72.5–74.5	67.5–74.5	67.5–70	69–75
35°C/72 h			(0–) 2–10 (−12)	11.5–12.5	13.5–16.5	5–12	6–12
Colony on SNA after 72 h (mm)
25°C/72 h		43–45	(32–) 37–47 (−51)	37.5–41.5	46.5–50.5	42.5–45	40–43
30°C/72 h			(15–) 32–54 (−64)	46.5–48.5	50.5–54.5	40–44	42–45
35°C/72 h			(0–) 1.5–6.2 (−14)	14.5–15.5	4.5–7.5	6–8	10–13
Colony on CMD after 72 h (mm)
25°C/72 h		58–60		77.5	59.5–61.5	77–79	72.5–74
30°C/72 h				70.5–72.5	77.5	55–62.5	60–62
35°C/72 h				11.5–12.5	7.5–10.5	no	no

**Figure 4:**
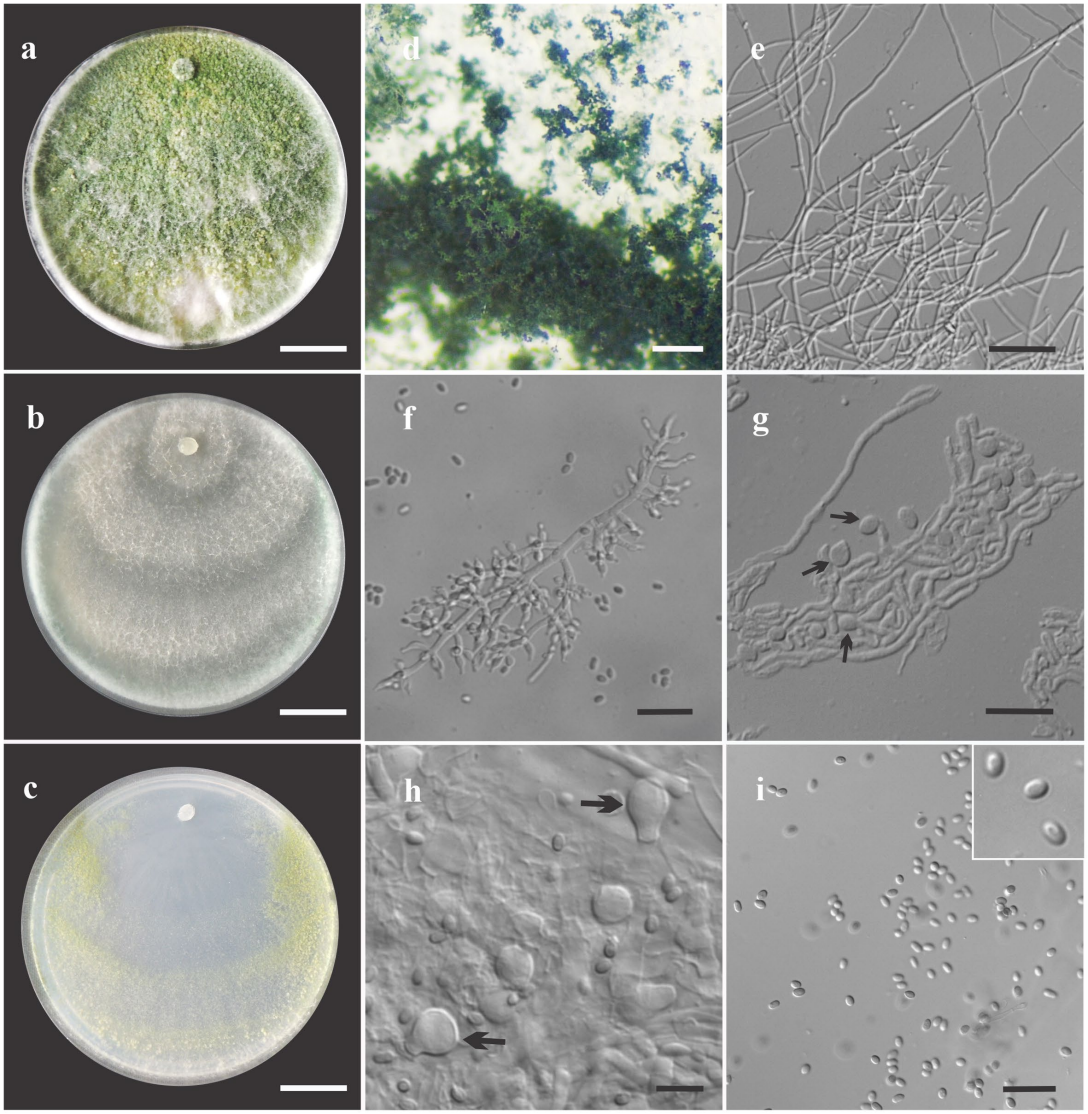
Asexual morph of *Trichoderma ararinum* sp. nov. from *Hevea brasiliensis* (VIC47509, holotype). (**A–C**) Colony appearances (PDA, CMD, and SNA; 7 days, 25°C). (**D**) Sporulating pustules (SNA). (**E**,**F**) Conidiophores and phialides (SNA). (**G**) Chlamydospore (after 48 h). (**H**) Chlamydospore (after 7 days). (**I**) Conidia. Scale bars: (**A–C**) 20 mm, (**D**) 0.2 mm, (**E**) 50 μm, (**F**,**G**,**I**) 20 μm, and (**H**) = 10 μm.

***Trichoderma ararianum*** V. N. Brito, J. L. Alves & M. V. Queiroz, **sp**. **nov**., Figure 4.

MycoBank MB830209

*Etymology*: “ararianum,” the specific epithet, refers to the river Arari from Itacoatiara, state of Manaus, Brazil.

*Holotype*: Brazil. State of Amazonas, Itacoatiara hydrographic basin, coordinates: 03°24′25.4″S, 058°29′38.8″W, V. N. Brito, 26 July 2017 (holotype VIC47509). Ex-type culture of COAD3345.

*Sexual morph* not seen. Colonies on PDA, SNA, and CMD fast growing at 25 and 30°C, with no growth at 35°C. The radial growth rate on CMD was 72.5–74 mm at 25°C and 60–62 mm at 30°C, after 72 h. Colonies covering plate after 4 days at 25°C, mycelia flat and thin, with a velvety texture, white, cottony, and raised. Chlamydospores globose to subglobose, terminal, intercalary, thick-walled, brown. Sporulation after 4 days, with dull green, fluffy tufts arranged in several concentric zones. Conidiophores with a central axis and short fertile branches or phialides growing along with it, additional branches coupled with internodes, and terminal part of conidiophore not branched. Phialides sparse, isolated; short side branches bearing terminal whorls of 2–3 phialides, slightly inclined or curved upward, 6–11 μm × 2–3.5 μm (*n* = 30), ampulliform, widest part mostly central, with a long neck. Conidia yellow-green, smooth, globose to subglobose, 2–5 μm × 2–3.5 μm (*n* = 30). On PDA, 80–82 mm at 25°C, 69–75 mm at 30°C, and 6–12 mm at 35°C, after 72 h. Colonies covering plate after 4 days at 25°C, mycelia dense and flat with floccose texture, mixed grayish yellow-green with dark herbage green hues due to sporulation; mycelia white and cottony in some areas of the colonies. The radial growth rate on SNA was 40–43 mm at 25°C, 42–45 mm at 30°C, and 10–13 mm at 35°C, after 72 h. Colonies covering the plate after 5 days at 25°C; mycelia white and thin. Sporulation unevenly spread in dull green areas. Chlamydospores globose to subglobose, terminal and intercalary, thick-walled, brown.

Habitat and host range: endophytic in the stems of living wild *Hevea brasiliensis* trees.

Additional specimens examined: Brazil. State of Amazonas, Itacoatiara hydrographic basin, coordinates 03°02′37,6″S, 058°30′11,8″W, V. N. Brito, 26 July 2017 (VIC47510, COAD3346); Brazil. State of Amazonas, Itacoatiara, coordinates 03°24′25.4″S, 058°29′38.8″W, V. N. Brito, 26 July 2017 (VIC47511, COAD3347); Brazil. State of Amazonas Itacoatiara, hydrographic basin, coordinates 03°24′25.4″S, 058°29′38.8″W, V. N. Brito, 26 July 2017 (VIC47512, COAD3348).

*Notes:* The concatenated tree with TEF1-α and *RPB*2 sequences clearly indicates that the four isolates belong to a new taxon (Figure 1). In addition, phylogenetic species were recognized based on two main previously accepted criteria Genealogical Concordance, wherein the clade (*T*. *ararianum*) was present in the two single-locus genealogies and Genealogical Non-Discordance, wherein the clade (*T*. *ararianum*) was well supported in the least one single-locus genealogy and was not contradicted in any other single-locus genealogy at the same level of support (Figure 2, Supplementary Figures 1, 2). *Trichoderma ararianum* sp. nov. is morphologically similar to several species of the *T*. *viride* clade and phylogenetically closely related to *Trichoderma koningiopsis* (Samuels et al., 2006). *Trichoderma ararianum* sp. nov. is distinct from *T*. *koningiopsis* based on; the faster colony growth rate on PDA at 25°C after 72 h (Table 3); the shorter, subglobose conidia, whereas *T*. *koningiopsis* has larger and broadly ellipsoidal conidia. In addition, the phialides are smaller, typically straight, lageniform, or slightly swollen in the middle, isolated and intercalary, or held in terminal whorls (with three); whereas *T*. *koningiopsis* has larger phialides, densely clustered with a very short internode and typically straight, though at times hooked or sinuous, narrowly lageniform held in whorls of 2–5, with several phialides at times arising from the same point and crowded (Table 3).

**Figure 5:**
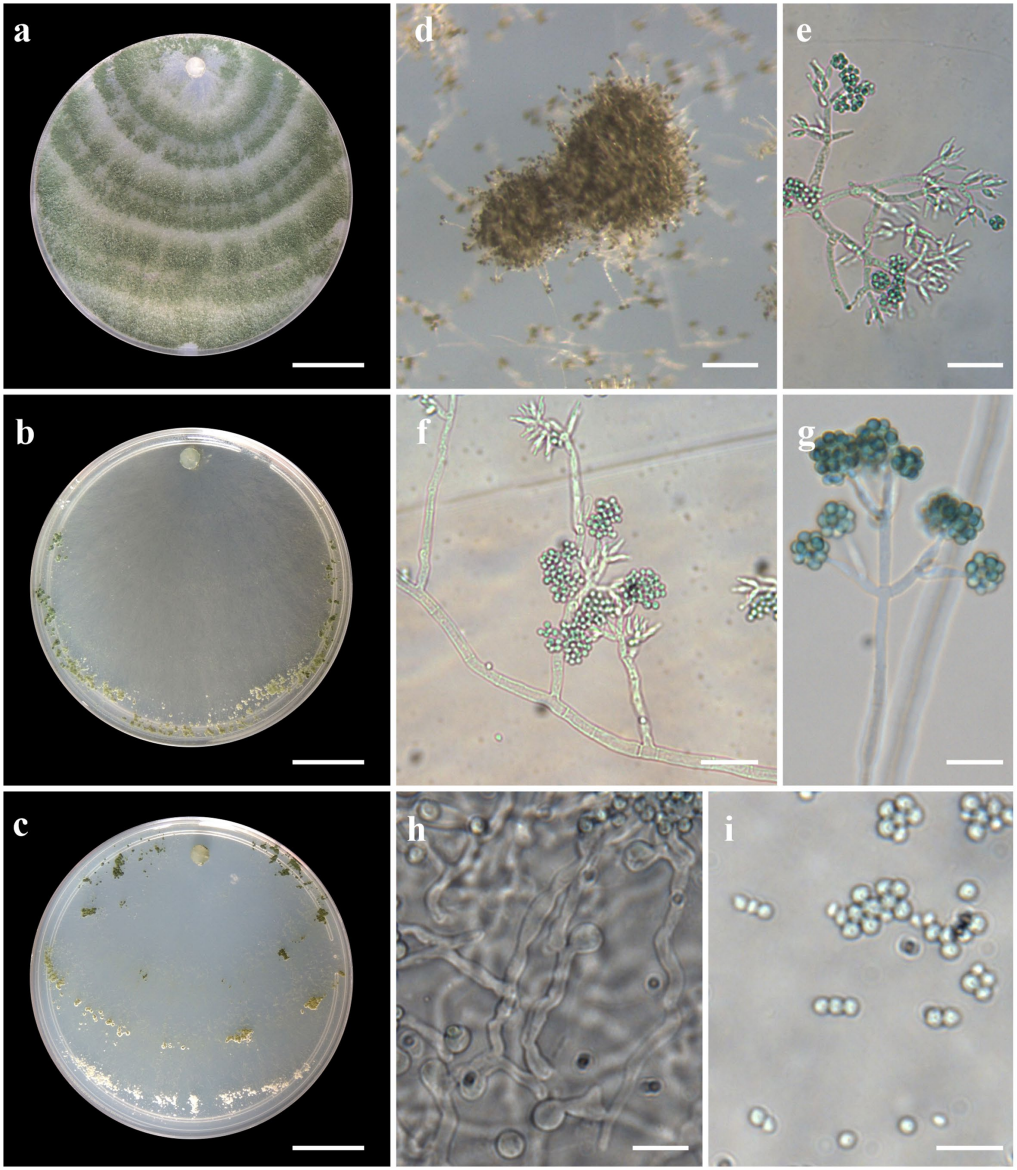
Asexual morph of *Trichoderma brasiliensis* sp. nov. from *Hevea brasiliensis* (VIC44363, holotype). (**A–C**) Colony appearances (PDA, CMD, and SNA; 7 days, 25°C). (**D**) Sporulating pustules (SNA). (**E–G**) Conidiophores and phialides (SNA). (**H**) Chlamydospore. (**I**) Conidia. Scale bars: (**A–C**) 20 mm, (**D**) 0.2 mm, (**E**,**F**,**H**) 20 μm, and (**G**,**I**) 10 μm.

***Trichoderma brasiliensis*** V. N. Brito & M. V. Queiroz, **sp**. **nov**., Figure 5.

MycoBank: MB830210

*Etymology*: “brasiliensis,” for its origin in *Hevea brasiliensis*.

*Holotype*: Brazil, State of Amazonas, Itacoatiara, coordinates 03°57′08.3″S, 59°05′16.8″W, V. N. Brito, 26 July 2017 (holotype VIC44363). Ex-type culture COAD2324.

*Sexual morph* not seen. Colonies with a fast growth rate at 30°C on all media with limited growth at 35°C. The radial growth rate on CMD was 59.5–61.5 mm at 25°C, 77.5 mm at 30°C, and 7.5–10.5 mm at 35°C, after 72 h. Colonies white, thin, with a downy surface. Chlamydospores globose, terminal, and intercalary, thick-walled, pale brown. Sporulation after 1 day, with dark green, fluffy tufts, or loose pustules arranged in several indistinctly separated, irregular concentric zones. Conidiophores consist of long main axes with pyramidal, verticillate branching in a pachybasium-type pattern (Kraus et al., 2004). Phialides 5–16 μm × 2–4 μm (*n* = 30), ampulliform to flask-shaped. Conidia green, smooth, globose to subglobose, 2–4 μm × 2–4 μm (*n* = 30). On PDA, after 72 h, 64.5–66.5 mm at 25°C, 67.5–74.5 mm at 30°C, and 13.5–16.5 mm at 35°C. Colonies covering the plate after 4 days at 25°C, mycelium dense and hairy. The radial growth rate on SNA was 46.5–50.5 mm at 25°C, 50.5–54.5 mm at 30°C, and 4.5–7.5 mm at 35°C, after 72 h. Colonies covered the plate after 6 days at 25°C; mycelium white and thin. Chlamydospores observed after 1 day; numerous, globose, grouping together in clusters, terminal and intercalary, thick-walled, brown. Sporulation starting after 2 days, arranged in several concentric zones spreading from the center and turning dark green.

Habitat and host range: In the roots of living wild *Hevea brasiliensis* trees.

Additional specimens examined: Brazil. State of Amazonas, Itacoatiara, coordinates 03°24′34.00″S, 058°29′18.3″W, V. N. Brito, 26 July 2017 (COAD2642); Brazil. Amazon state, Itacoatiara hydrographic basin, coordinates 03°24′34.00″S, 058°29′18.3″W, V. N. Brito, 26 July 2017 (COAD2643); Brazil. State of Amazonas, Itacoatiara, coordinates 03°24′34.00”S, 058°29′18.3”W, V. N. Brito, 26 July 2017 (COAD2644).

*Notes:* Phylogenetic analysis clearly indicates that the three isolates belong to a new taxon, with robust distinction in multilocus trees (Figure 1), the GCPSR concept (Figure 2), and single-locus trees (Supplementary Figures 1, 2). *Trichoderma brasiliensis* sp. nov. shares morphological similarities with *Trichoderma brevicompactum* (Kraus et al., 2004), such as ampulliform phialides arising in crowded verticils, however, the conidia of *T*. *brevicompactum* are subglobose to short ellipsoidal while those of *T*. *brasiliensis* sp. nov. are globose to subglobose.

**Figure 6:**
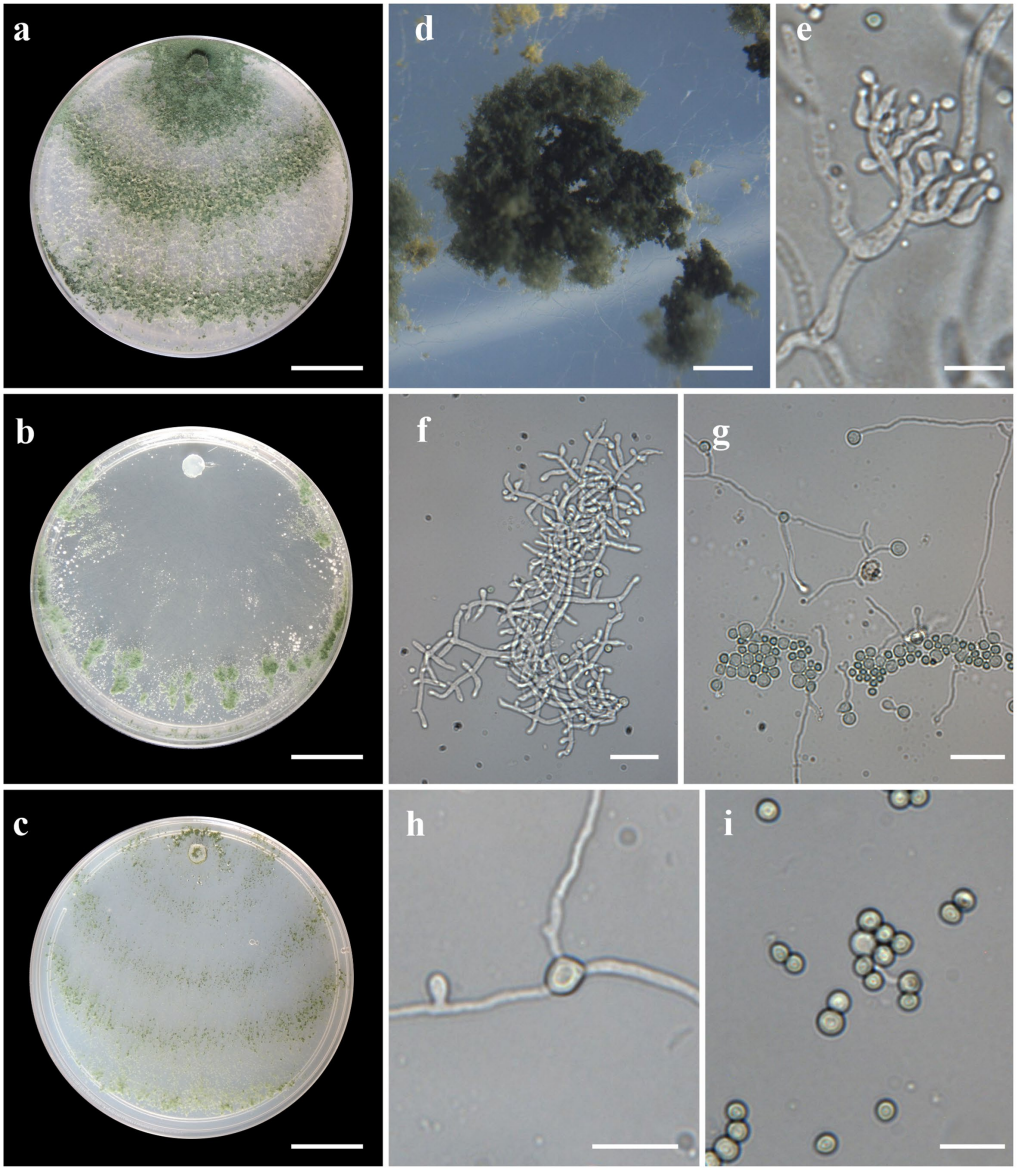
Asexual morph of *Trichoderma heveae* sp. nov. from *Hevea* spp. (VIC44362, holotype). (**A–C**) Colony appearances (PDA, CMD, and SNA; 7 days, 25°C). (**D**) Sporulating pustules (SNA). (**E,F**) Conidiophores and phialides (SNA). (**G**,**H**) Chlamydospores. (**I**) Conidia. Scale bars: (**A–C**) 20 mm, (**D**) 0.5 mm, (**E**,**I**) 10 μm, and (**F–H**) 20 μm.

***Trichoderma heveae*** V. N. Brito & M. V. Queiroz, **sp**. **nov**., Figure 6.

MycoBank: MB830211

*Etymology*: Latin, *heveae*, refers to the host from which the fungus was isolated.

*Holotype*: Brazil, State of Amazonas, Itacoatiara, coordinates 7°44′04.1″S/72°49′51.2″W, V. N. Brito, 26 July 2017 (holotype VIC44362). Ex-type culture: COAD2323.

*Sexual morph* not seen. Colonies with a rapid growth rate at 25°C on all media with limited growth at 35°C. The radial growth rate on CMD was 77.5 mm at 25°C, 70.5–72.5 mm at 30°C, and 11.5–12.5 mm at 35°C, after 72 h. Colonies thin, surface downy, floccose or farinose, yellowish-green to green. Chlamydospores globose, terminal, and intercalary. Sporulation starting after 2 days; dark green, fluffy tufts, or loose pustules, with irregular or circular outlines, arranged in several indistinctly separated concentric zones. Conidiophores formed along with main axes, with long branches at lower levels, and sparse, widely spaced, solitary or paired phialides; or short, one-celled side branches bearing terminal whorls of 2–3 phialides, slightly inclined or curved upward, 4–12 μm × 2–4 μm, (*n* = 30), ampulliform, widest part mostly central. Conidia green, smooth, subglobose or oval, 2–4 μm × 2–4 μm (*n* = 30). The radial growth rate on PDA was 77.5 mm at 25°C, 72.5–74.5 mm at 30°C, and 11.5–12.5 mm at 35°C, after 72 h. Mycelium dense and hairy covering the plate after 3 days at 25°C. Radial growth rate on SNA was 37.5–41.5 mm at 25°C, 46.5–48.5 mm at 30°C, and 14.5–15.5 mm at 35°C, after 72 h. Mycelium covering the plate after 5 days at 25°C; colonies hyaline and thin. Chlamydospores formed after 2 days; globose, terminal, intercalary, thick-walled, brown. Sporulation starting after 2 days, arranged in several concentric zones spreading from the center and turning dark green.

Habitat and host range: In the stems (COAD2646) and leaves (COAD2645 and COAD2323) of living, wild *Hevea brasiliensis* trees.

Additional specimens examined: Brazil. State of Amazonas, Manaus, Coari hydrographic basin, coordinates 03°45′27.4″S, 063°24′34.6″W, V. N. Brito, 26 July 2017 (COAD2645); Brazil. State of Amazonas, Manaus, Itacoatiara, hydrographic basin, coordinates 03°02′40.4″S, 058°30′09.7″W, V. N. Brito, 26 July 2017 (COAD2646).

*Notes:* Phylogenetic analysis clearly indicates that the three isolates represent a new taxon, with robust distinction in multilocus trees (Figure 1), the GCPSR concept (Figure 2), and single-locus trees (Supplementary Figures 1, 2). *Trichoderma heveae* sp. nov. is a tropical endophytic species present in the leaves and stems of wild *Hevea*; belonging to the *Viride* clade (Figure 1). *Trichoderma subviride* Qin & Zhuang, a closely related species, has slower growth rates on all media (CMD 58–60 mm, PDA 60–63 mm, and SNA 43–45 mm; Qin and Zhuang, 2016b), while *T*. *heveae* sp. nov. colony showed a fast growth rate in the media (CMD 77.5 mm, PDA 77.5 mm, and SNA 37.5–41.5 mm; Table 3).

## Discussion

In recent years, there has been growing interest in the isolation and identification of endophytic *Trichoderma* spp. and their derived products (Contreras-Cornejo et al., 2016; Katoch et al., 2019; Nuankeaw et al., 2020), and a number of new species, such as *T*. *amazonicum* (Chaverri et al., 2010), *T*. *spirale* (Chaverri et al., 2003), *Trichoderma protrudens* (Samuels & Chaverri; Degenkolb et al., 2008), and *Trichoderma botryosum* (Rodriguez et al., 2021), have been described. In the present study, 30 endophytic isolates of the genus *Trichoderma* were obtained from the leaves, stems, and roots of wild rubber trees in the Brazilian Amazon region, with four new species being identified, based on morphological and molecular analyses.

Cai and Druzhinina (2021) reported that at least 60% of *Trichoderma* species have precise and accurate molecular identification at the species level based on an analysis of three DNA barcodes (ITS, TEF1-α, and *RPB*2). Other studies have recently confirmed that TEF1-α and *RPB*2 gene sequences well support taxonomic conclusions in *Trichoderma* due to their suitable interspecific variations (Jaklitsch and Voglmayr, 2015; Qiao et al., 2018; Gu et al., 2020; Rodriguez et al., 2021). Furthermore, Cai and Druzhinina (2021) reported that *Trichoderma* species can be identified based on pairwise similarities between *RPB*2 and TEF1-α. In this study, single and combined TEF1-α and *RPB*2 datasets were used for molecular analysis to reveal the phylogenetic relationships between the species.

The *Viride* and *Harzianum* clades had the highest percentage of isolates. In addition, isolates belonging to the *Virens*, *Strictipile*, and *Brevicompactum* clades were identified, demonstrating that endophytic species in tropical climates are distributed over different clades of the genus *Trichoderma*, as demonstrated by Zhang et al. (2007) and Gazis and Chaverri (2010).

*Trichoderma* species were categorized into five clades, with a prevalence of rubber-tree isolates in the *Viride* clade, represented by 63% of isolates obtained in this study. The *Viride* clade is among the largest in *Trichoderma*, and its taxonomy has been examined in detail over the past decades, revealing a number of new species (Jaklitsch et al., 2006; Qin and Zhuang, 2016b). This is a cosmopolitan clade containing both saprotrophic and endophytic species, reportedly found in *Theobroma cacao* L., *Theobroma gileri* Cuatrec., and *H*. *brasiliensis* (Samuels et al., 2006; Gazis and Chaverri, 2010). Among the species of the *Viride* clade, *T*. *ovalisporum* has been reported as an endophyte in *Theobroma* spp. (Evans et al., 2003; Holmes et al., 2004), which has been found in both Southeast Asia and South America (Samuels et al., 2006).

Another related species within the *Viride* clade is *T*. *atroviride*, which occurs in Neotropical regions, as well as in Europe, colonizing tree bark or living as a mycoparasite (Hoyos-Carvajal et al., 2009; Jaklitsch, 2011). *Trichoderma atroviride* has also been isolated as an endophyte from the ornamental trees *Cephalotaxus fortunei* Hook. and *Camptotheca acuminata* Decne. in China (Zheng et al., 2011; Pu et al., 2013).

*Trichoderma koningiopsis* is a cosmopolitan species that has been recorded mainly from the Neotropics. Its *Hypocrea* sexual morph has been found as far north as the State of New York (Samuels et al., 2006), although the sexual form was not found in this study. Evidence suggests that *T*. *koningiopsis* is effective in plant protection, and it has been used in biological control applications. In addition, it has been reported as an endophyte of both cultivated and wild *Coffea canephora* Pierre ex Froehner in Cameroon (Rodriguez et al., 2021).

In this study, three new species belonging to the *Viride* clade were identified. Among them, two species are close to *T*. *koningiopsis* and the other is related to *T*. *subviride*. *Trichoderma acreanum* sp. nov. and *T*. *ararianum* sp. nov. are different from *T*. *koningiopsis*, as is evident from both phylograms (Figures 1, 2 and Supplementary Figures 1, 2). Additional morphological data show that the new taxa vary in the size and shape of the conidia and phialides, as well as in their growth rates on several media. *Trichoderma heveae* sp. nov. is different from *T*. *subviride*, as evidenced by both phylograms (Figures 1, 2 and Supplementary Figures 1, 2). *Trichoderma subviride* is a common wood-inhabiting species, while *T*. *heveae* sp. nov. has been identified as an endophyte in the leaves and stems of *Hevea* spp. in the Amazon Forest. Although these species have different ecological niches, their morphological characteristics are similar.

*Trichoderma brasiliensis* sp. nov. is most closely related to *T*. *brevicompactum*, an endophytic species from North America, South America, and Asia. However, *T*. *brevicompactum* has only been reported as an endophyte in garlic (*Allium sativum* L.) in China (Shentu et al., 2014) and not in tropical tree species. The *Brevicompactum* clade originally comprised *Trichoderma arundinaceum* (Zafari, Gräfenhan & Samuels), *Trichoderma brevicompactum*, *Trichoderma protrudens*, *Trichoderma rodmanii* (Samuels and Chaverri; Jaklitsch and Voglmayr, 2013) and *Trichoderma turrialbense* (Samuels, Degenkolb, Nielsen and Gräfenhan; Degenkolb et al., 2008). *Trichoderma aurantioeffusum* (Jaklitsch) and *Trichoderma margaretense* (Jaklitsch) were added later (Jaklitsch, 2011), likewise that *Trichoderma grande* (Qin & Zhuang) and *Trichoderma limonium* (Qin and Zhuang, 2016a). Samuels et al. (1998) comprehensively studied this section and emphasized the importance of asexual features and sequence data (TEF1-α and *RPB*2) in its taxonomy. Recently, several species have been described based on their phenotypic and genotypic differences. The species in the *Brevicompactum* clade are characterized by a conidiophore type known as “pachybasium,” with small and compact phialides (Bissett, 1991; Kraus et al., 2004). The new species related to this clade displayed similar characteristics, including the size, shape, and color of the conidia (Table 3). However, *T*. *brasiliensis* sp. nov. has larger and less aggregated phialides compared with *T*. *brevicompactum*.

The *Harzianum* clade of *Trichoderma* comprises species associated with a wide variety of substrates. However, this clade is characterized by a “species complex” and is morphologically heterogeneous and phylogenetically complex (Chaverri et al., 2015). Several morphologically similar species are known to be phylogenetically related to *T*. *harzianum*. In our study, five isolates grouped in the *Harzianum* clade, together with *T*. *azevedoi*, *T*. *breve*, and *T*. *lentiforme*. The taxonomic complex of the *Harzianum* clade includes combinations of different geographic origins and hosts (Rodriguez et al., 2021; Zheng et al., 2021), of ecological and economic importance, with promising prospects for use in biological control (Chaverri et al., 2015).

*Trichoderma virens* has a worldwide distribution, mainly in soil, and is recently reported as an endophyte in the aerial tissues of wild coffee in Africa (Rodriguez et al., 2021). In our study, one isolate (84F15C–AM) obtained from a stem of *H*. *brasiliensis* is phylogenetically close to *T*. *virens* and grouped with two isolates within the *Virens* clade.

Considering the significance of *Trichoderma* spp. in biocontrol, Baiyee et al. (2019) reported the mechanisms involved in the biological control of pathogens in lettuce (*Lactuca sativa* L.) by which *T*. *spirale* T76-1 inhibits the severity of leaf spot disease. In the present study, *T*. *spirale* belonging to the *Strictipile* clade was isolated from the stems of *H*. *brasiliensis*, and this endophytic characteristic may provide additional benefits.

In conclusion, our results indicate the presence of eight endophytic species of the genus *Trichoderma* previously reported in other studies (*T*. *atroviride*, *T*. *erinaceum*, *T*. *koningiopsis*, *T*. *lentiforme*, *T*. *ovalisporum*, *T*. *sparsum*, *T*. *spirale*, and *T*. *virens*), as well as four new endophytic species (*T*. *acreanum* sp. nov., *T*. *ararianum* sp. nov., *T*. *heveae* sp. nov., and *T*. *brasiliensis* sp. nov.) isolated from *Hevea* spp. native to the Amazon Forest. From the small sample size, it is obvious that wild rubber trees harbor an array of endophytic *Trichoderma* species with the potential for use in the management of rubber diseases, as well as bioprospecting for bioactive compounds.

## Data availability statement

The datasets presented in this study can be found in online repositories. The names of the repository/repositories and accession number(s) can be found in the article/Supplementary material.

## Author contributions

MQ designed the project, supervised its execution, and reviewed and edited the manuscript. VB contributed to the overall data analysis and wrote the first draft of the manuscript. VB, JL, KS, and TS contributed to the preparation of phylogenetic trees. VB and KS contributed to the collection of material, isolation, identification, and preservation of fungal isolates. VB, KS, and TS contributed to the standardization of the endophytic fungal isolation technique and sequence analysis. VB, KS, and CB contributed to DNA extraction, PCR, and gene sequencing. VB, KS, and JL contributed to morphological and molecular characterization. VB, KS, TS, OL, JL, and MQ interpreted the bioinformatics analysis. All authors contributed to the article and approved the submitted version.

## Funding

This research was supported by the Coordenação de Aperfeiçoamento de Pessoal de Nível Superior (CAPES)—Finance Code 001, the Conselho Nacional de Desenvolvimento Científico e Tecnológico (CNPq), and the Fundação de Amparo à Pesquisa do Estado de Minas Gerais (FAPEMIG).

## Conflict of interest

The authors declare that the research was conducted in the absence of any commercial or financial relationships that could be construed as a potential conflict of interest.

## Publisher’s note

All claims expressed in this article are solely those of the authors and do not necessarily represent those of their affiliated organizations, or those of the publisher, the editors and the reviewers. Any product that may be evaluated in this article, or claim that may be made by its manufacturer, is not guaranteed or endorsed by the publisher.

## Footnotes

^1^http://dna.macrogen.com

^2^http://www.DnaBaser.com

^3^http://tree.bio.ed.ac.uk/sofware/fgtree/

^4^https://github.com/b-brankovics/GCPSR
