# Supplementary figures and images for: Endophytic Trichoderma species from rubber trees native to the Brazilian Amazon, including four new species
Source: Front Microbiol. 2023 Apr 18;14:1095199. doi: 10.3389/fmicb.2023.1095199 (PMC10151590; doi:10.3389/fmicb.2023.1095199)

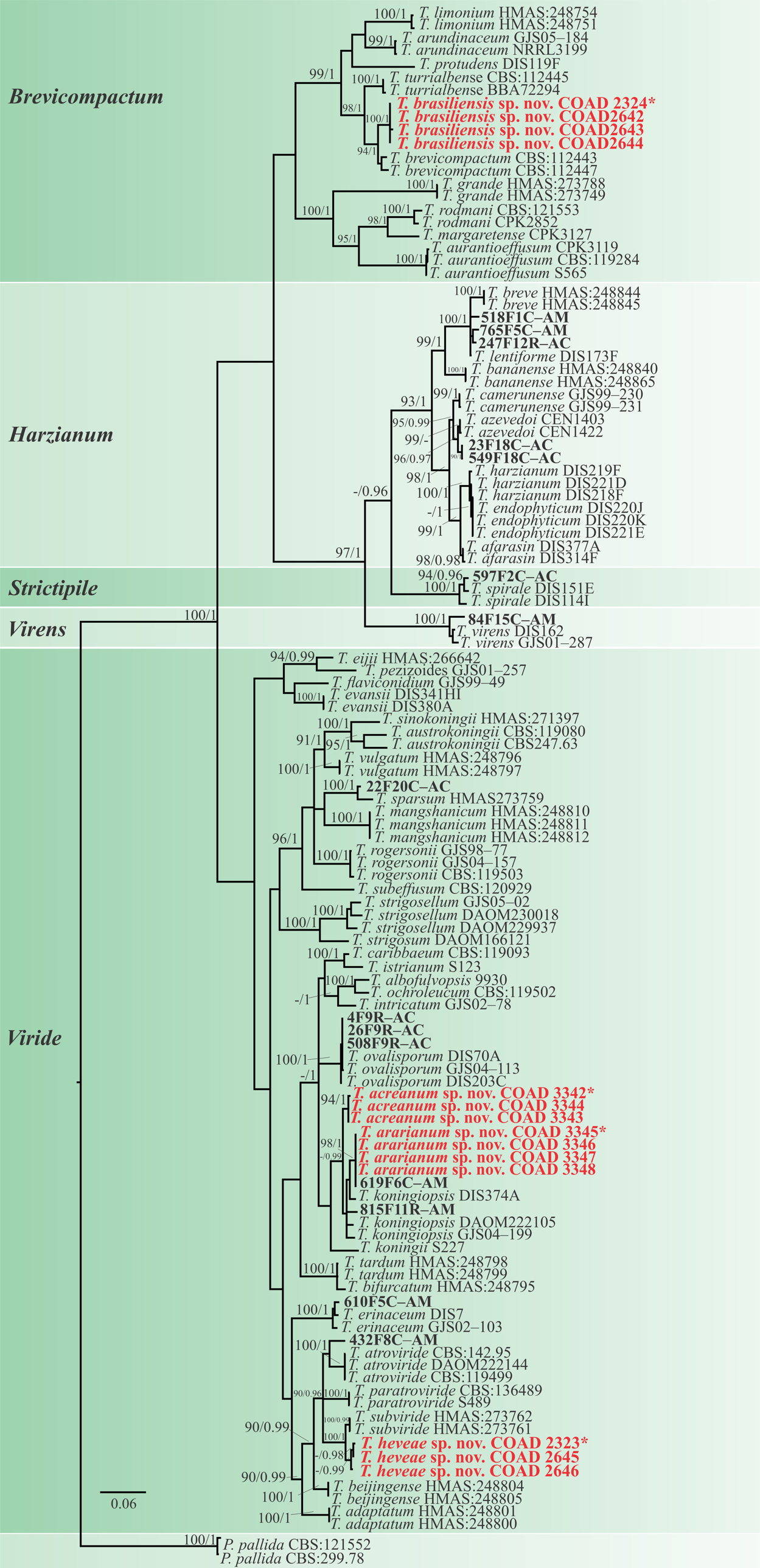

Supplement: Supplementary file 2 [file Image_1.JPEG]

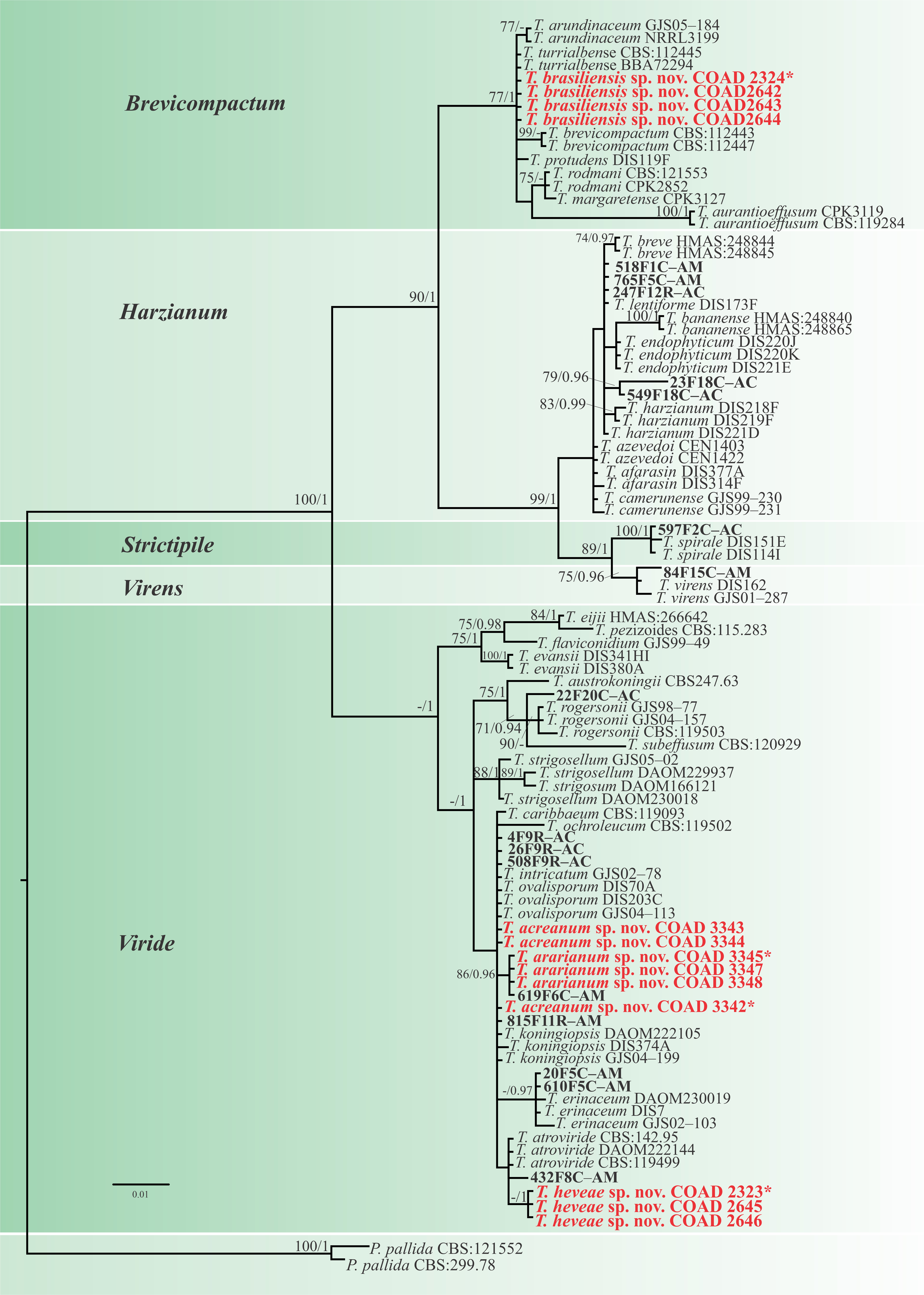

Supplement: Supplementary file 4 [file Image_3.JPEG]
